# Via-less electromagnetic band-gap-enabled antenna based on textile material for wearable applications

**DOI:** 10.1371/journal.pone.0246057

**Published:** 2021-01-28

**Authors:** Adel Y. I. Ashyap, N. I. M. Elamin, S. H. Dahlan, Z. Z. Abidin, Chan Hwang See, H. A. Majid, Najib AL-Fadhali, Jameel A. A. Mukred, Gameel Saleh, B. A. F. Esmail

**Affiliations:** 1 Center for Applied Electromagnetic (EMCenter), Faculty of Electrical and Electronic Engineering, Universiti Tun Hussein Onn Malaysia, (UTHM) Batu Pahat, Johor, Malaysia; 2 Faculty of Electronics and Electrical Engineering, International University of Africa, Khartoum, Sudan; 3 Advanced Telecommunication Research Center (ATRC), Faculty of Electrical and Electronic Engineering, Universiti Tun Hussein Onn Malaysia, (UTHM) Batu Pahat, Johor, Malaysia; 4 School of Engineering and the Built Environment, Edinburgh Napier University, Edinburgh, United Kingdom; 5 Universiti Tun Hussein Onn Malaysia, Pagoh Education Hub, Pagoh, Muar, Johor, Malaysia; 6 Department of Biomedical Engineering, College of Engineering, Imam Abdulrahman Bin Faisal University, Dammam, Saudi Arabia; Information Technology University, PAKISTAN

## Abstract

A compact fabric antenna structure integrated with electromagnetic bandgap structures (EBGs) covering the desired frequency spectrum between 2.36 GHz and 2.40 GHz for Medical Body-Area Networks (MBANs), is introduced. The needs of flexible system applications, the antenna is preferably low-profile, compact, directive, and robust to the human body's loading effect have to be satisfied. The EBGs are attractive solutions for such requirements and provide efficient performance. In contrast to earlier documented EBG backed antenna designs, the proposed EBG behaved as shielding from the antenna to the human body, reduced the size, and acted as a radiator. The EBGs reduce the frequency detuning due to the human body and decrease the back radiation, improving the antenna efficiency. The proposed antenna system has an overall dimension of 46×46×2.4 mm^3^. The computed and experimental results achieved a gain of 7.2 dBi, a Front to Back Ratio (FBR) of 12.2 dB, and an efficiency of 74.8%, respectively. The Specific Absorption Rate (SAR) demonstrates a reduction of more than 95% compared to the antenna without EBGs. Moreover, the antenna performance robustness to human body loading and bending is also studied experimentally. Hence, the integrated antenna-EBG is a suitable candidate for many wearable applications, including healthcare devices and related applications.

## 1. Introduction

Over the past decade, the rapid development in communication systems for body sensor networks (BSN) has grown notably, supporting several applications ranging from patient monitoring systems, tracking, and emergency rescue systems to personalized health care systems and battlefield survival [1–4]. Many wireless standards were proposed for commercialization and research to support these growing developments, including the Medical Implant Communication Systems, Industrial, Scientific, and Medical (ISM). More recently, the Federal Communication Commission (FCC) introduced the MBANs frequency band covering the 2.36 GHz to 2.4 GHz spectrum due to its clean spectrum and low interference sources [5].

Improvements in efficiency and effectiveness of flexible/wearable antennas have become essential in light of these advances in Wireless Body Area Network (WBAN) application. An additional essential requirement for flexible/wearable antennas than traditional designs is to reduce the interaction between the radiating elements and the body tissue, despite being close to each other. It is noted that prolonged exposure to human tissue is controversial because it may exhibit health hazard effects [6]. To address this issue, consideration should be given to study the effects of flexible antennas on human tissue, such as the maximum allowable Specific Absorption Rate (SAR) [7–11]. Furthermore, to introduce lightweight, conformal, and low profile features [12, 13] into the antenna design criteria have further posed considerable challenges to meet the design's specification. The EBGs are proposed to divert the wearable antenna radiation from the human tissues. However, the previously reported structures with the integrated antenna in the area of wearable design have several disadvantages such as (i) the design could be too thick or electrically large to be suitable for the wearable systems [14–24], (ii) the design may be printed on semi-flexible materials which cannot withstand several bends [5, 12, 25–27] and (iii) the design may reveal poor Front-to-Back ratio (FBR) [28–31].

In this paper, a fully textile antenna with novel EBG for WBAN applications at 2.4 GHz is proposed. This work lays on the bandgap feature (Dispersion Diagram Technique) of EBG that cannot be demonstrated by earlier reported wearable EBG structures [8, 11, 12, 14–16, 18, 19, 22, 23, 25]. The bandgap feature is controlled by vias, such as in the case of mushroom-like EBG. It also may be achieved by a complex structure that can increase the effective inductance, such as multi-layered EBG, meander line EBG, fractal EBG, and interdigitated EBG. However, if the vias or complex structure is used, the risk of manufacturing inaccuracies is greater since the used material is fabric. Thus, designing EBG from fabric materials with a bandgap feature is challenging. Therefore, a simple and novel structure based on fabric materials that increased the effective inductance which introduces the bandgap feature is presented. The size is also reduced compared to several reported fabric EBG at 2.4 GHz [14–31].

In contrast to earlier documented EBG backed antenna designs, the proposed EBG behaved not only as a ground plane for shielding the antenna from the body but also as a radiator and reduced the reference antenna size. The proposed design contributes to enhancing the FBR, increasing the gain, and reducing the antenna's SAR level significantly.

It is worth to mention that compared with the lately recorded wearable antennas functioning in the super wideband [32], UWB [33], and UHF frequency bands [34], this article mainly emphasizes the realization of low profile, lightweight, flexible, and narrow-band wearable antenna for off-body communication in WBANs.

The paper is organized as follows. The physical structures of the EBG are presented, and the features are explained in Section 2. The experimental investigations and analysis of the integrated antenna with the EBG are discussed in Section 3. Section 4 presents the proposed antenna's performance in terms of the SAR value and the bending effect. Finally, conclusions are drawn in Section 5.

## 2. Methodology of the antenna and EBG design

### 2.1 Antenna design

The fabric material with a permittivity of 1.7 and a thickness of 0.7 mm is used as a substrate. Simultaneously, a ShieldIt^TM^ material with a thickness of 0.17 mm is used for the conducting elements.

**Fig 1:**
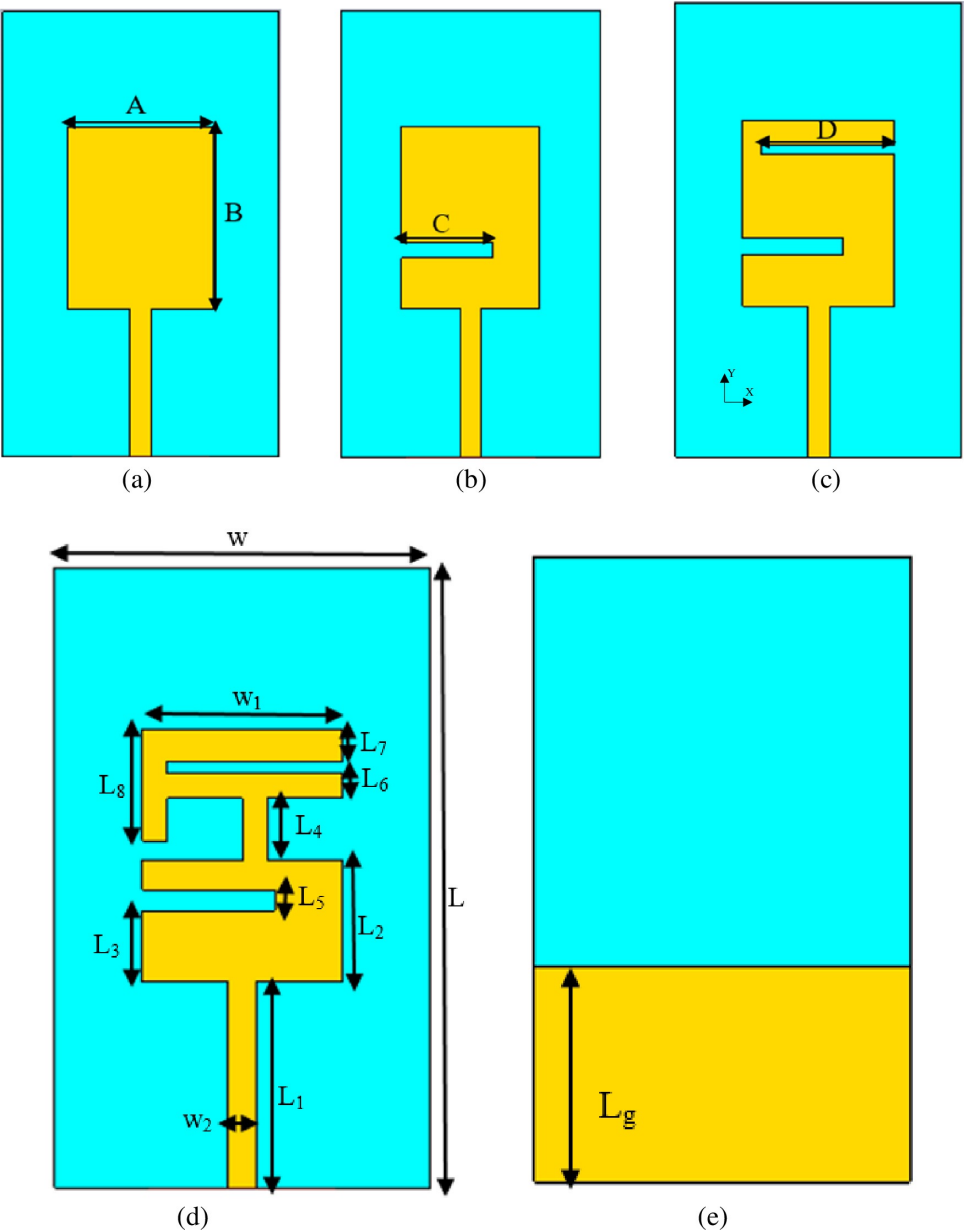
Configuration of the reference antenna design (a) 1^st^ design, (b) 2^nd^ design, (c) 3^rd^ design, (d) 4^th^ design (reference antenna), and (e) Back view for all designs.

**Fig 2:**
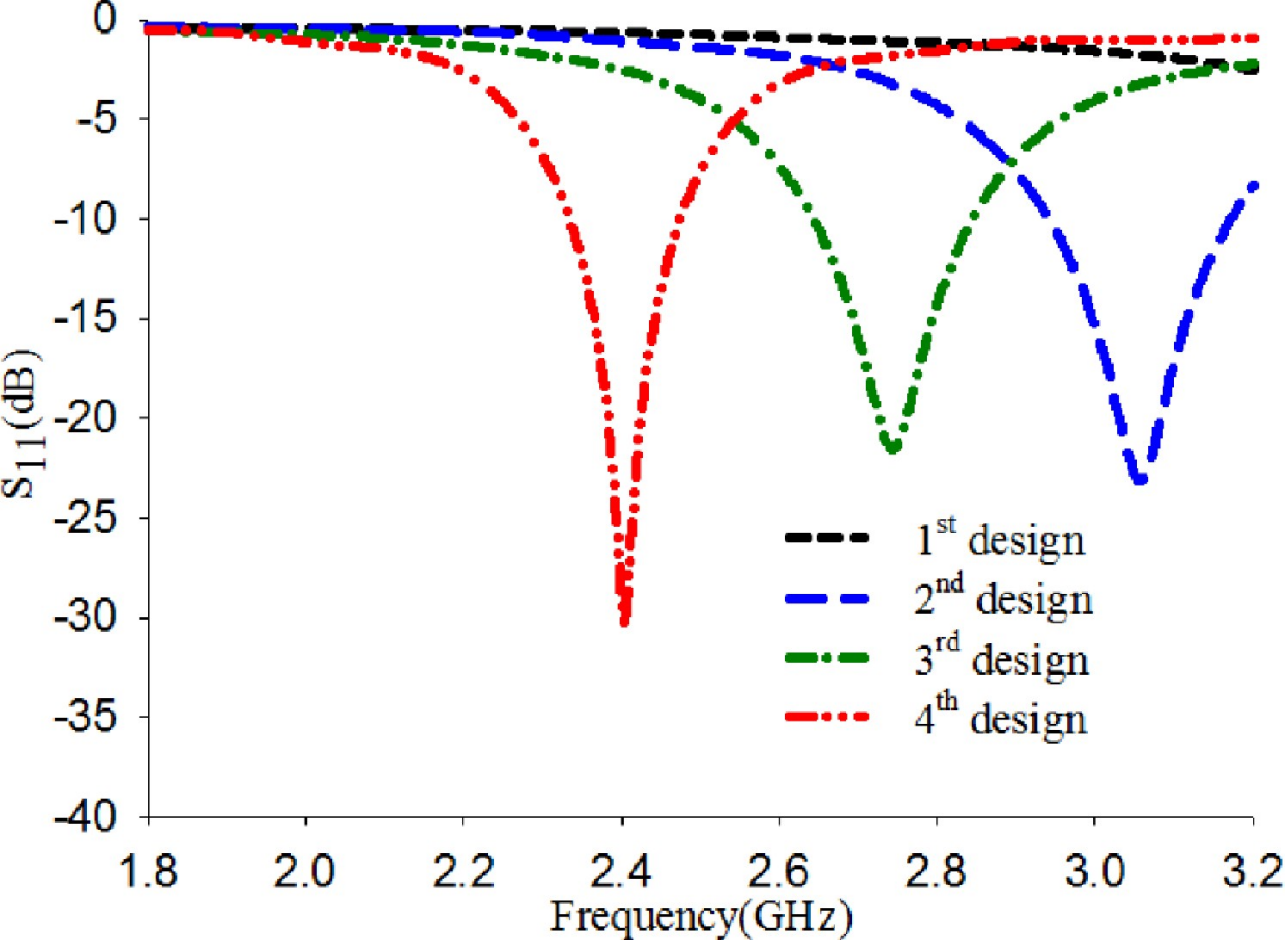
S_11_ of the evolution designs.

The antenna is designed based on the combination of F-shaped and U-shaped connected by a stripline. These shapes are formed by introducing specific slots and connected by stripline. The selection of the line widths, the slot lengths, and their positions are based on parametric studies. The design's evolution process is depicted in Fig 1, and their S_11_ is presented in Fig 2. In all design processes, the ground plane is fixed with L_g_ = 17.4 mm. The dimension of line widths and the slot lengths is presented in Table 1. Fig 2 shows the impact of introducing slots on the design. It can be seen that as the slots were introduced, the resonant frequency shifted to the lower band. The slots meandered, exciting the patch's surface current and lowered the antenna's resonance frequency to the desired frequency. The overall dimension of the finalized proposed design is 50×30×0.7 mm^3^.

**Table 1:** Optimal values of the reference antenna dimensions (mm).

Parameter	A	B	C	D	w	w_1_	w_2_	L	L_1_	L_2_	L_3_	L_4_	L_5_	L_6_	L_7_	L_8_	L_g_
Value	16	20.4	10.6	14	30	16	2.295	50	16.6	9.8	5.7	4.97	1.65	2	2.65	9	17.4

### 2.2 EBG design

**Fig 3:**
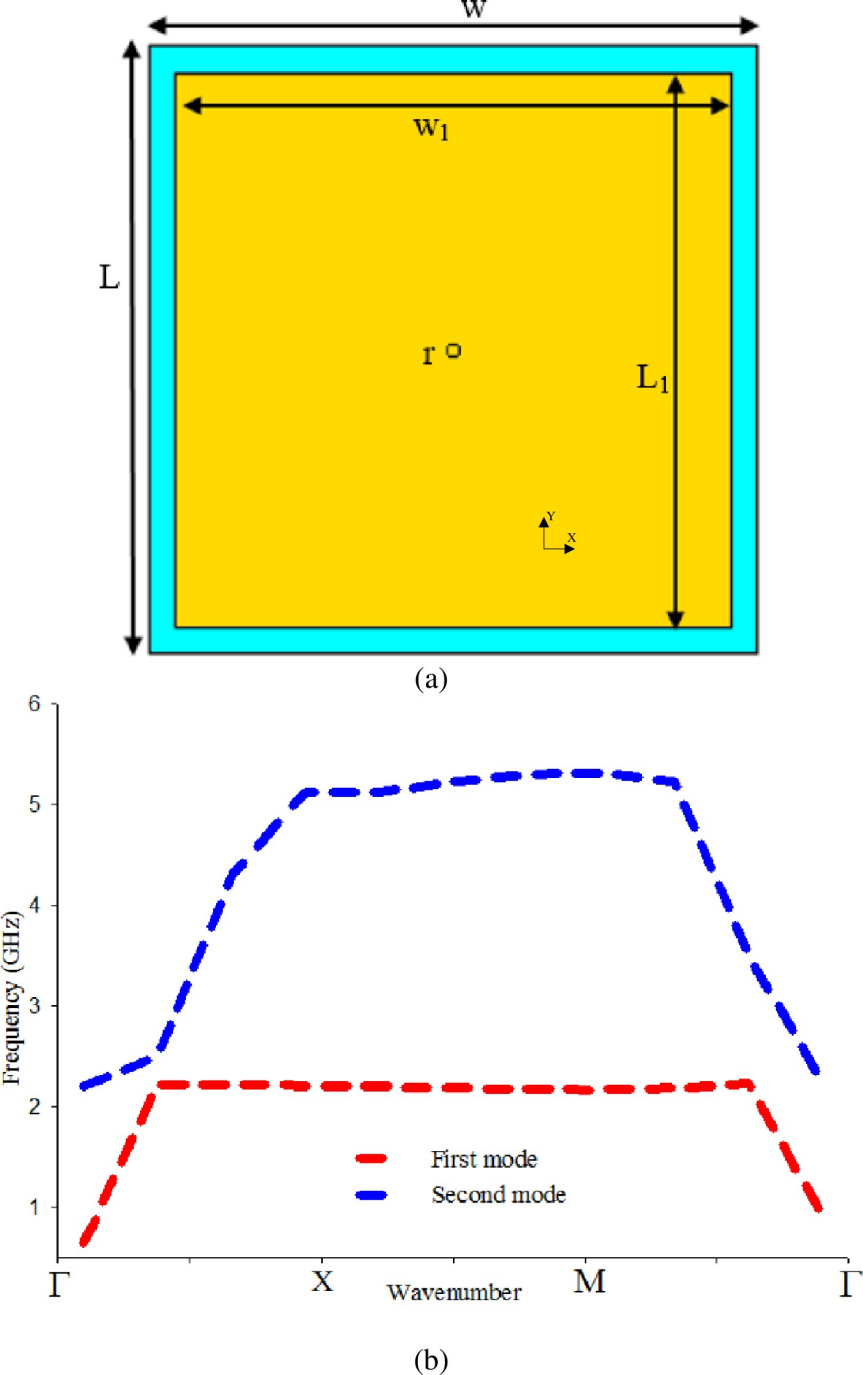
(a) Mushroom-like EBG unit cell, and (b) Bandgap characteristics of (a).

**Fig 4:**
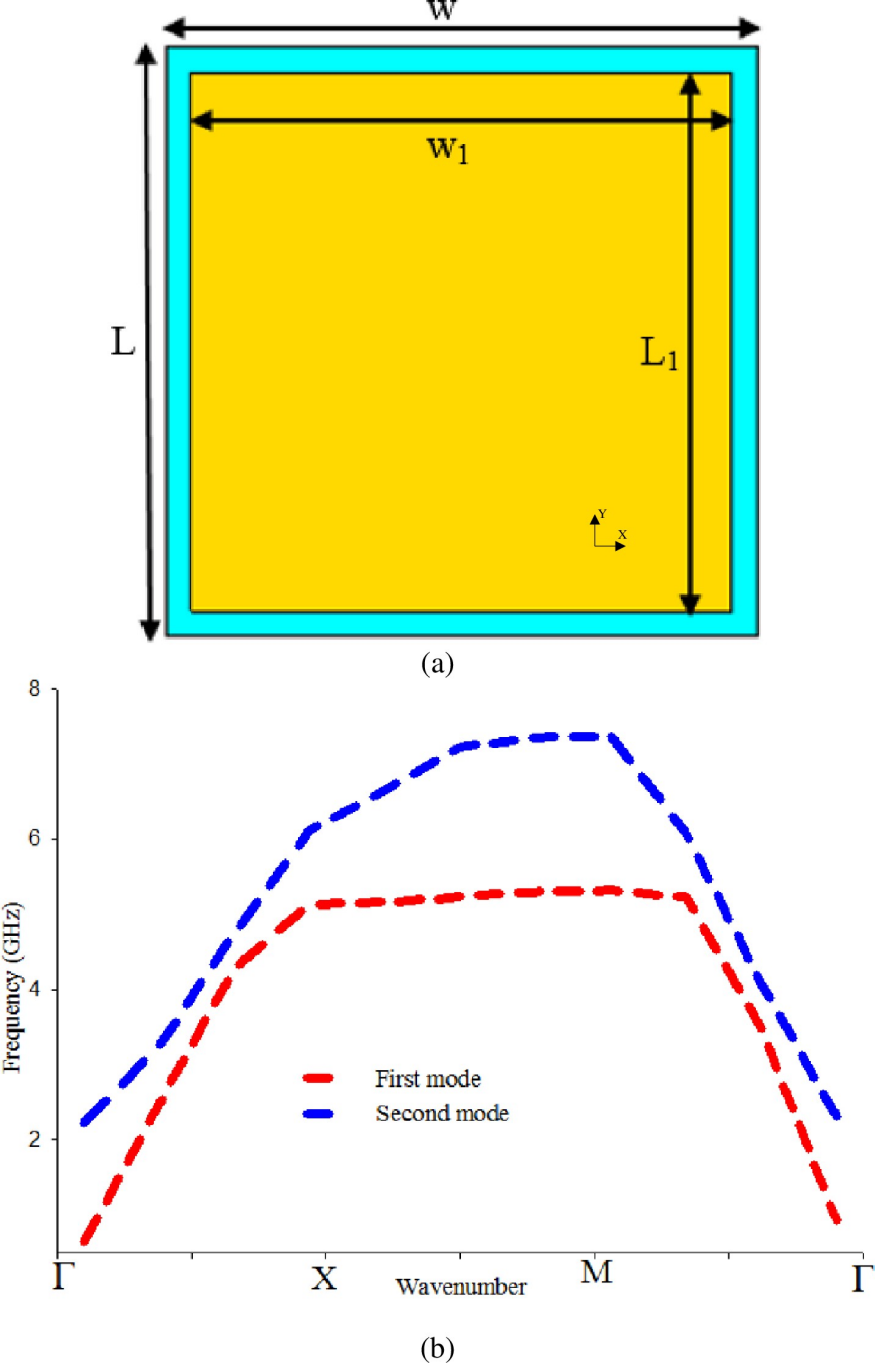
Vialess square patch EBG unit cell, and (b) Bandgap characteristics of (a).

The EBG unit cell structure evolved from a conventional mushroom-like EBG, as depicted in Fig 3(A). The dispersion diagram technique is used to analyze the EBG unit cell [35]. The desired bandgap is obtained with an overall dimension of 23×23×0.7 mm^3^, as shown in Fig 3(B). However, the existence of vias while using fabric material makes the process of fabrication more complicated. Therefore, vias are removed and becomes vialess (uni-planar). The vialess unit cell with the same dimension of exciting vias as depicted in Fig 4(A) is simulated, but the obtained bandgap does not cover the desired band 2.4 GHz as shown in Fig 4(B). The absence of vias caused the bandgap to shift to higher frequency bands, as mention in [36].

**Fig 5:**
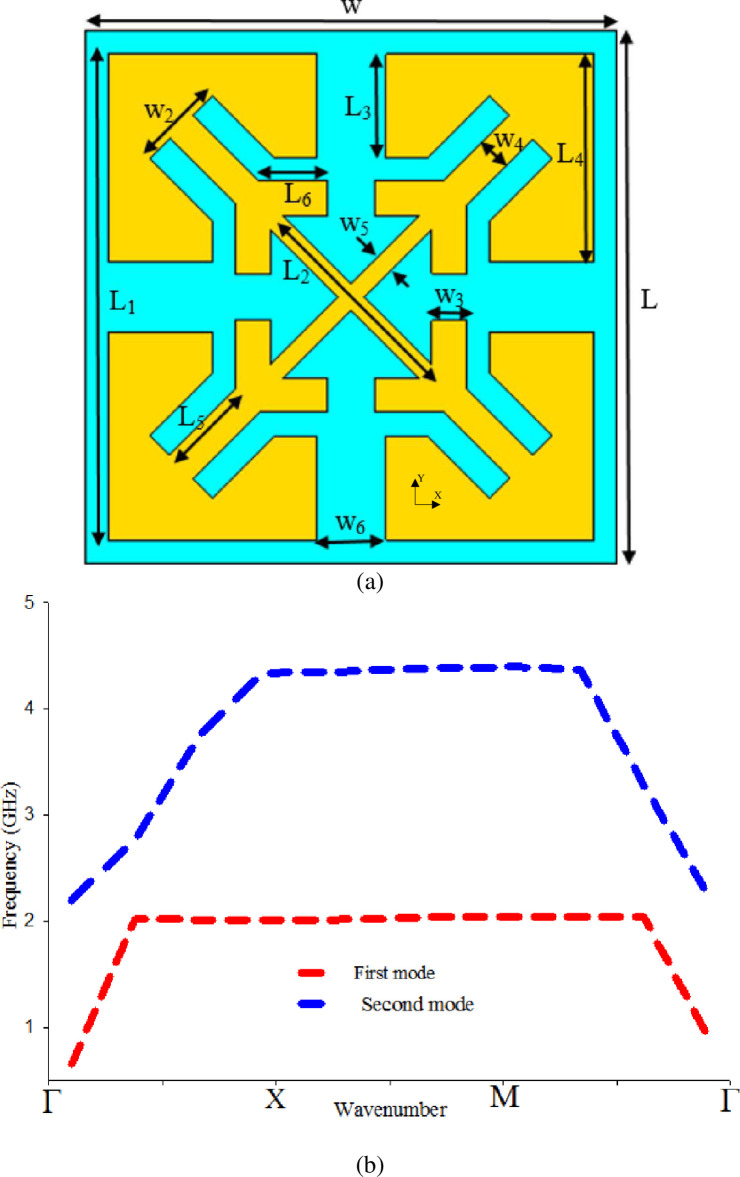
(a) Vialess proposed EBG unit, and (b) Band-gap characteristics of the proposed vialess EBG unit cell.

A significant benefit of the “uni-planar” EBG design is the elimination of the vertical via. This makes the fabrication process easier and compatible with millimeter-wave and microwave circuits. However, the complex EBG structure possesses its challenges in prototyping even if vias have been eliminated. Producing this from fabric materials makes the task even more difficult and might lead to inaccuracy. One of the motivations is to minimize design complexity while gaining maximum output from the EBG. Hence, the vialess EBG unit cell is modified as depicted in Fig 5(A). The proposed design reveals the desired bandgap cover of 2.4 GHz, as presented in Fig 5(B). Without the existing on the vias, the proposed crossed strip lines could increase the inductance [37] and control the bandgap with a simple structure that can be easily fabricated using fabric materials. The optimum dimensions are tabulated in Table 2.

**Table 2:** Optimal values of the proposed EBG dimensions (mm).

Parameter	w	w_1_	w_2_	w_3_	w_4_	w_5_	w_6_	r	L	L_1_	L_2_	L_3_	L_4_	L_5_	L_6_
Value	23	21	3.8	1.5	1.4	1	3	0.25	23	21	10	4.5	9	4	3

### 2.3 Integration of antenna with EBG

**Fig 6:**
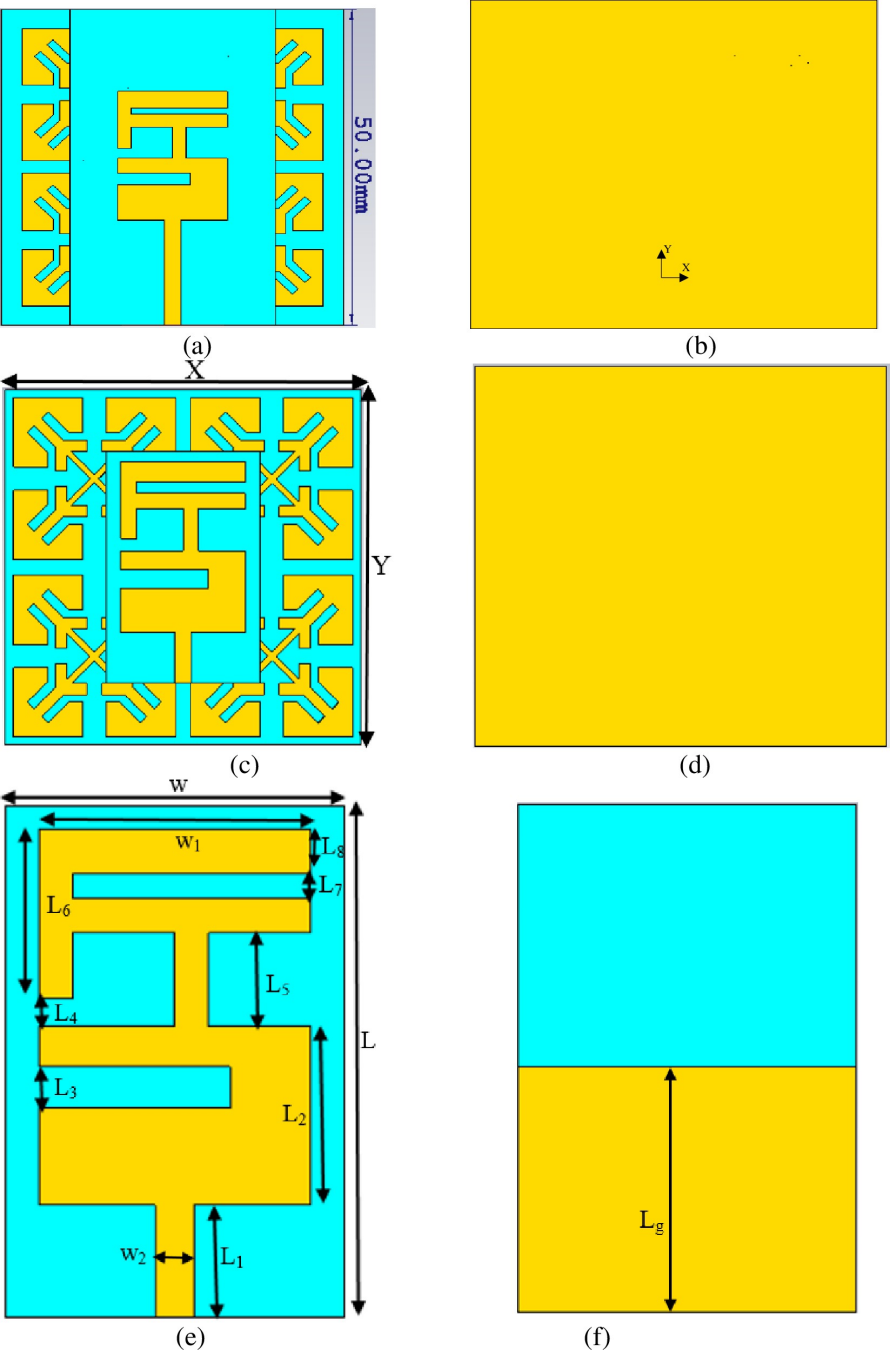
(a) Front view of the reference antenna with EBG, (b) Back view of the reference antenna with EBG, (c) Front view of the modified antenna with EBG, (d) Back view of the modified antenna with EBG, (e) Front view of Modified antenna, and (f) Back view of Modified antenna.

**Fig 7:**
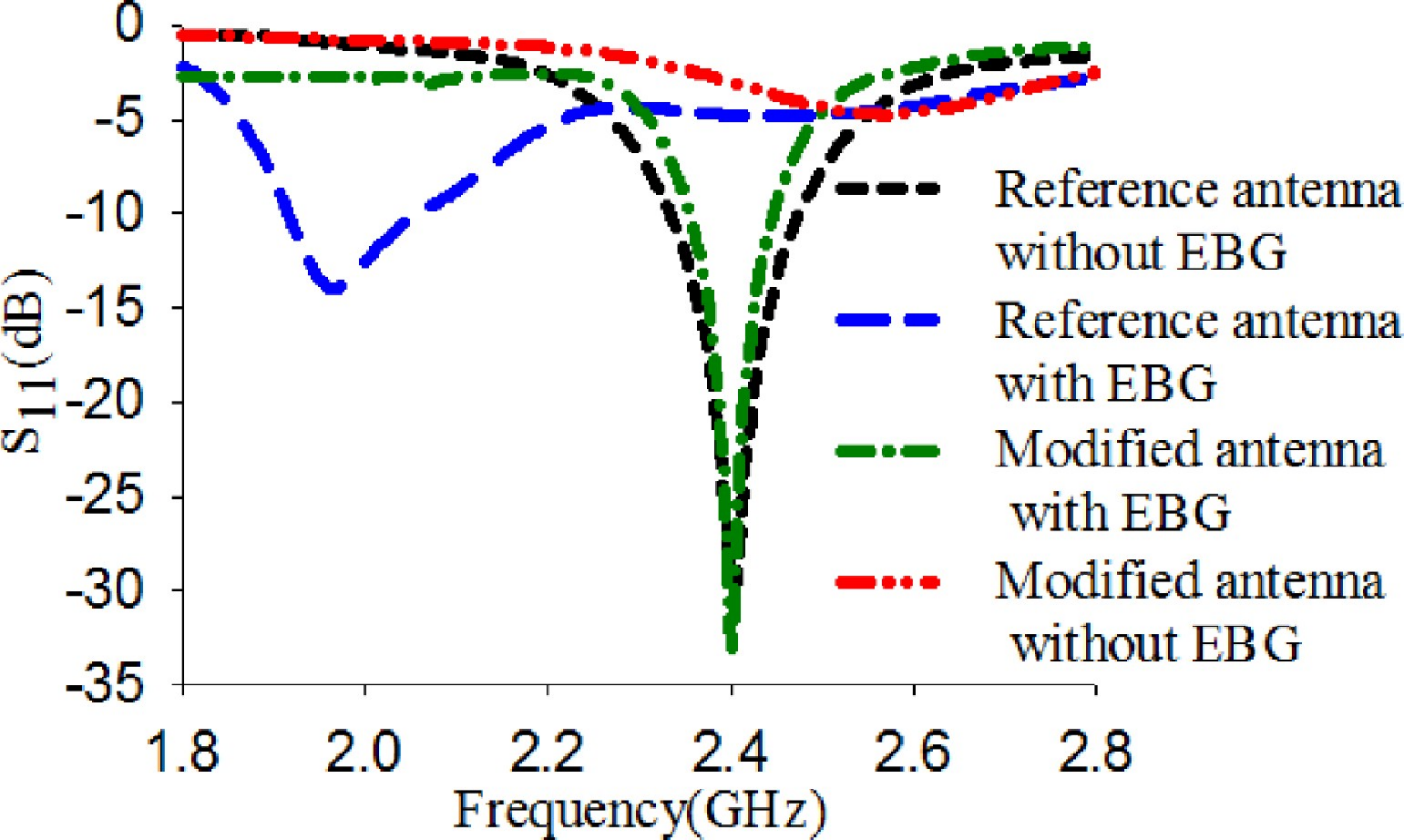
S_11_ of the reference antenna alone, the integrated reference antenna with EBG, the modified antenna with EBG, and the modified antenna alone.

The reference antenna was placed on a 2×2 EBG array, as revealed in Fig 6(A). A 1 mm foam layer is inserted between the two layers to prevent any connection that causes a short circuit. The overall size of the integrated antenna with EBG, including the foam, is 50×50×2.4 mm^3^. The S_11_ of reference antenna with and without EBG is depicted in Fig 7. It is seen that the reference antenna alone shows an operating frequency covered the 2.4 GHz. However, adding EBG to the reference antenna revealed that the S_11_ is shifted from 2.4 GHz to 1.96 GHz. In order to obtain the desired band covering 2.4 GHz, the reference antenna and the EBG ground plane dimensions are modified as shown in Fig 6(C), 6(E) and 6(F). The optimum dimensions are tabulated in Table 3. This modification shows that the modified antenna's size can be reduced by 60% compared to the reference antenna.

**Table 3:** Optimal values of the modified antenna dimensions (mm).

Parameter	w	w_1_	w_2_	L	L_1_	L_2_	L_3_	L_4_
Value	20	16	2.295	30	6.6	10.5	2.4	1.6
Parameter	L_5_	L_6_	L_7_	L_8_	L_g_	X	Y	
Value	5.475	10	1.47	2.65	14.5	46	46	

**Fig 8:**
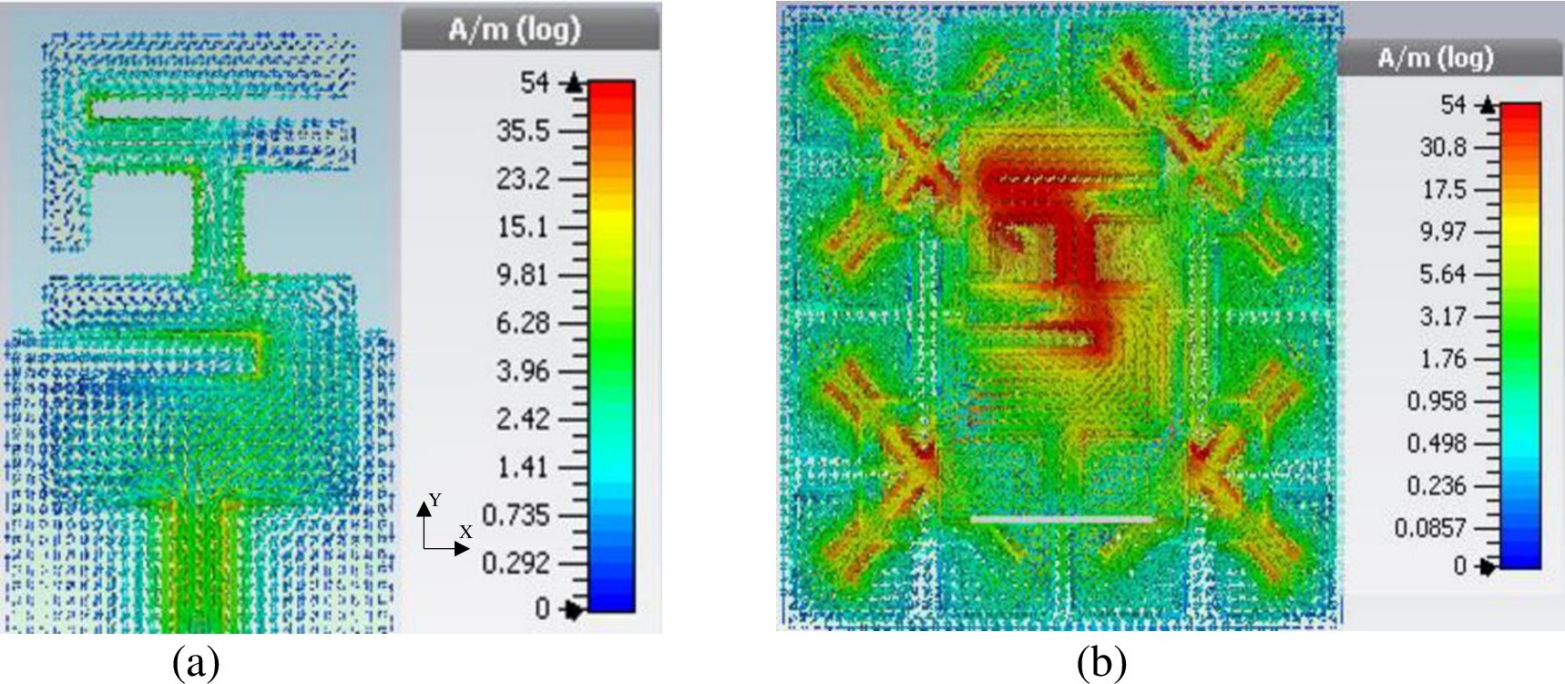
Surface current (a) Modified antenna alone, (b) Reference antenna, and (c) Modified antenna with EBG.

To further confirm the role of the EBG in reducing the size of the reference antenna, the surface current is simulated at 2.4 GHz for the modified antenna alone, and the modified antenna with EBG as illustrated in Fig 8. The results in Fig 8(A) show that when the modified antenna is alone, less current is concentrated on the radiating patch. This is due to the modified antenna alone does not resonate at 2.4 GHz. However, the modified antenna's surface current with EBG shows a maximum concentrated current density mainly located on the radiating elements. This observation indicates that EBG could contribute as a radiator and reduce the size of the reference antenna.

## 3. Performance of antenna and EBG in free space

### 3.1 Normal case (flat)

**Fig 9:**
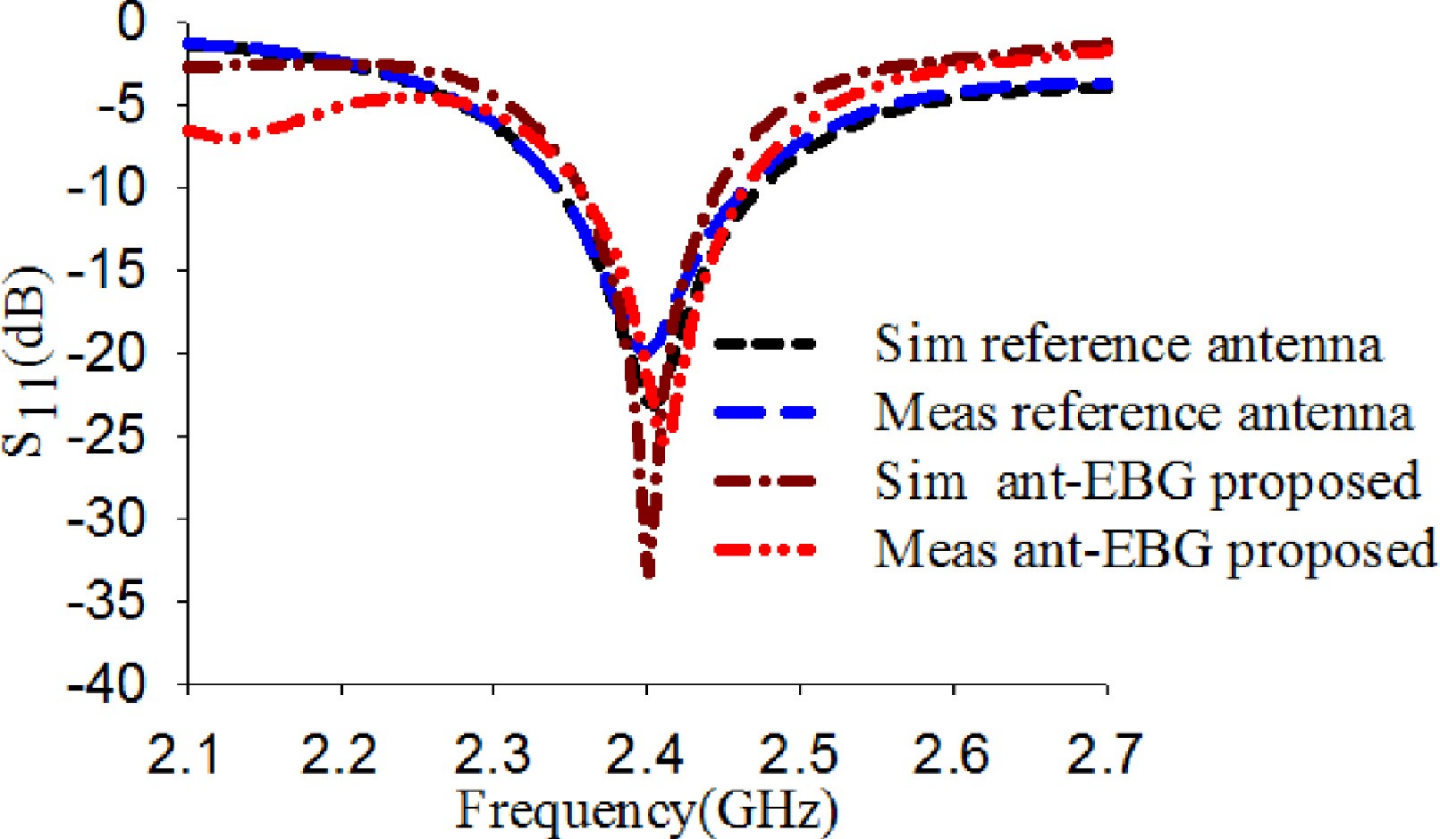
S_11_ result of the reference antenna alone, and integrated modified antenna with EBG.

The reference antenna and the modified antenna with EBG were modeled, and their performance was predicted using CST Microwave Studio [38]. Fig 9 describes the simulated and measured S_11_ results of the reference antenna and the modified antenna with EBG. This demonstrates that the measured results are in good agreement with the simulated results. Very slight differences can be noticed, and these may be attributed to fabrication errors and soldering tolerance.

**Fig 10:**
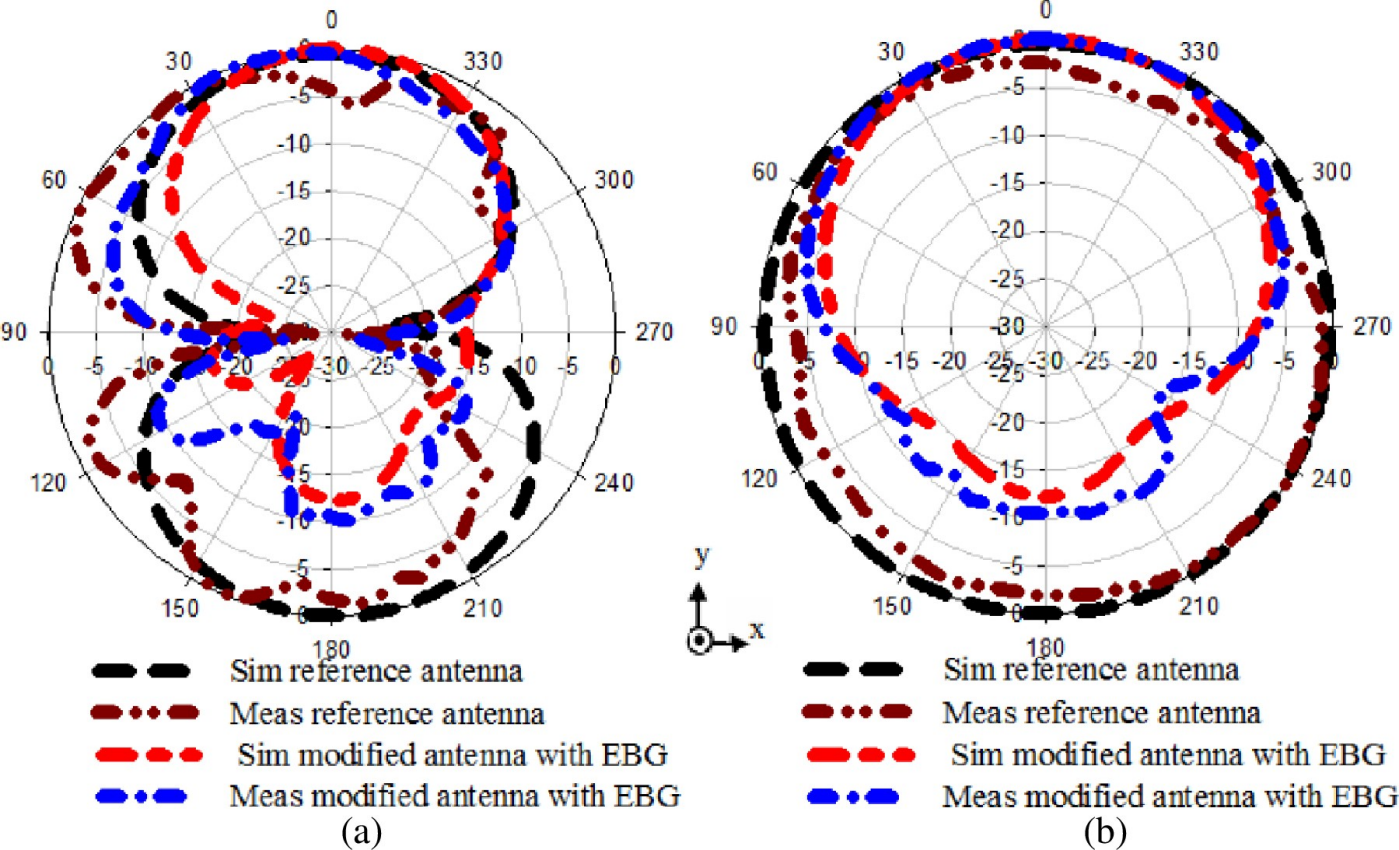
Normalized radiation performance of the reference antenna alone, and integrated modified antenna with EBG vialess (a) x-z plane, and (b) y-z plane.

Fig 10 presents the normalized numerical and experimental radiation patterns of the reference antenna and the modified antenna with EBG along the x-z and y-z plane at 2.4 GHz. It can be seen that the reference antenna along the x-z plane has maximum radiation in the ±z-direction while an omnidirectional along the y-z plane. The maximum radiation in the negative z-direction is not preferred for wearable antennas operating close to the body. This is due to the radiation towards the body that may present a health risk factor when it is irradiated over prolonged periods [15]. A directional radiation pattern is preferred when the antenna operates with other devices far away from the body. However, the modified antenna with EBG shows directional radiation by reflecting the back lobe toward the +z-direction along both planes x-z and y-z. As a result, the radiation towards the body is reduced by 12.3 dB. Furthermore, the modified antenna shows a better gain of 7.2 dBi than the reference antenna with a gain of 2.2 dBi. Also, it reveals an efficiency of 74.8%.

### 3.2 Deform case

**Fig 11:**
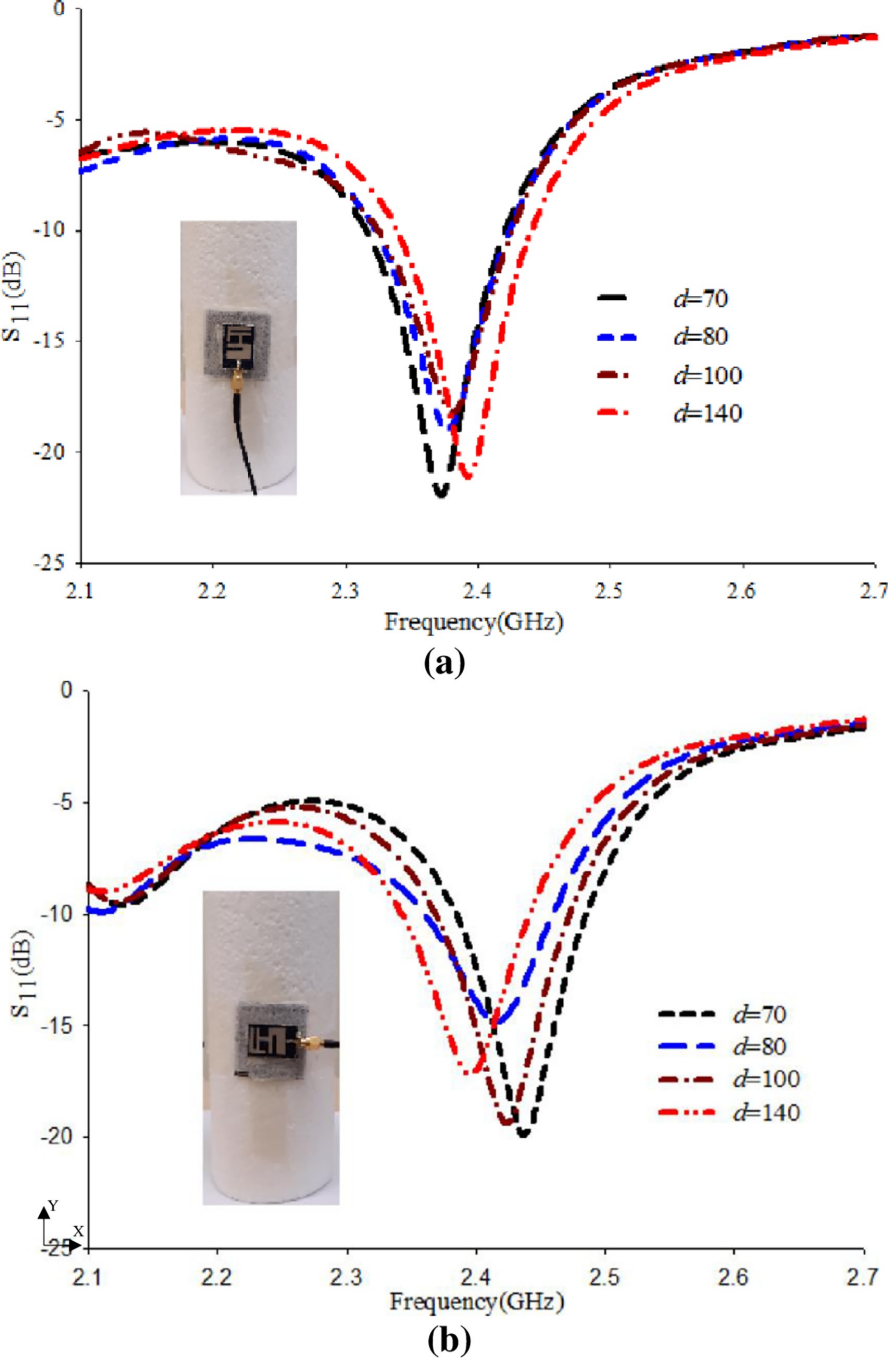
S_11_ performance based on bending along (a) x-z plane, and (b) y-z plane.

The bending analysis of the proposed antenna over foam cylinder along the x-z plane and y-z plane for varying diameters (70 mm, 80 mm, 100 mm, 140 mm) were experimentally carried out to ensure that the performance under bending condition still satisfies the wearable application. In this study, the diameters were chosen to approximate the arm and leg of the human body. The results are depicted in Fig 11. It shows that the bending has nearly no effect on the modified antenna with EBG performance. It is realized that although the operating frequency band and the resonance frequency are slightly shifted, the S_11_ still maintains the bandwidth covering the 2.4 GHz ISM band when the bending diameter (*d*) is varied in the range from 70 to 140 mm.

**Fig 12:**
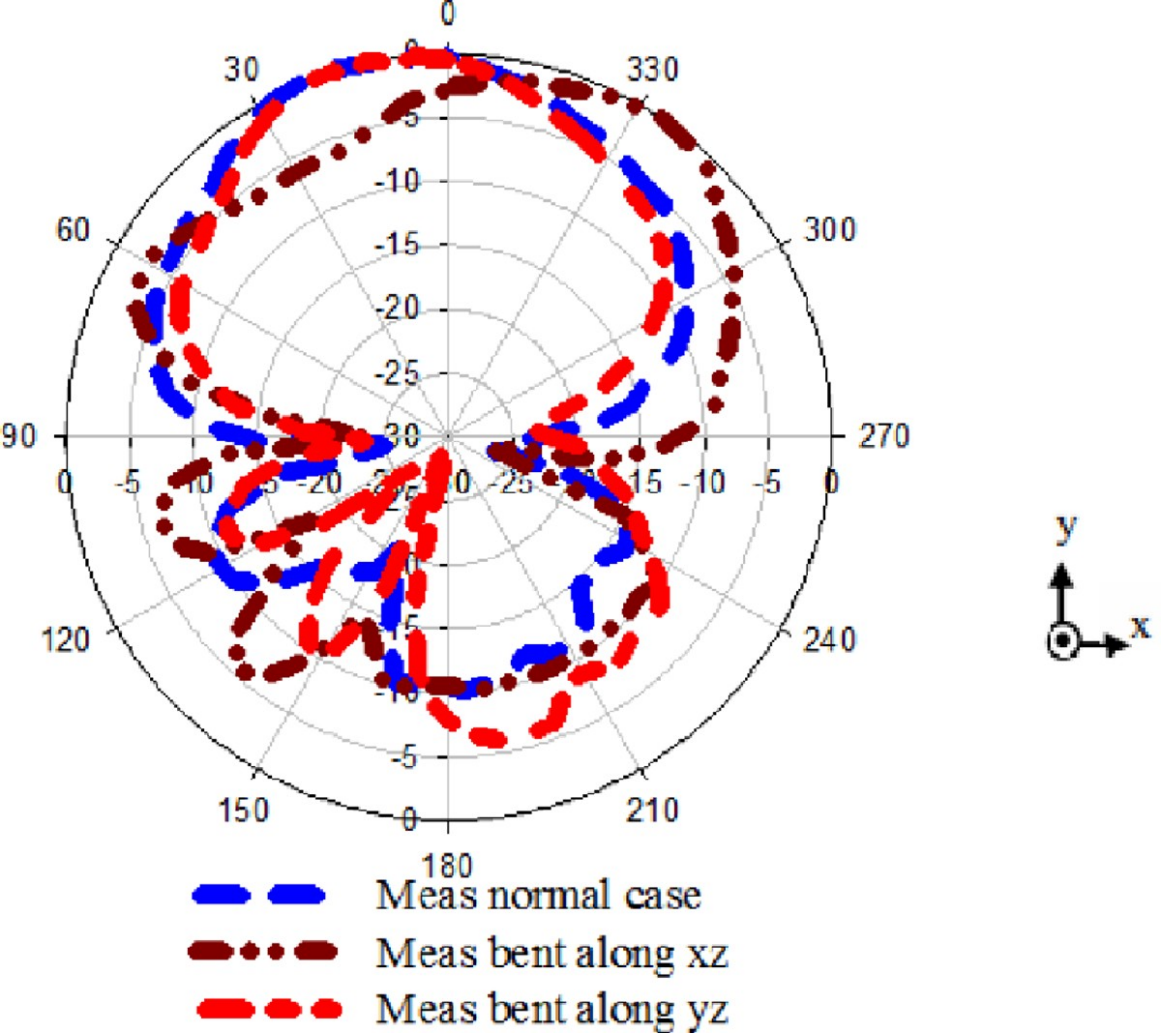
Radiation pattern performance based on bending at diameter d = 140 along x-z and y-z planes.

The modified antenna's normalized radiation pattern performance with EBG was also experimentally investigated along x-z and y-z plane with a diameter of *d* = 140 mm. The radiation patterns are tested at 2.4 GHz, as revealed in Fig 12, and demonstrates acceptable radiation patterns with good gain around 6.4 dBi, FBR around 8 dBi, and efficiency around 66%. This investigation demonstrates that our structure is highly conformal and a suitable candidate for body-worn devices.

## 4. Ethical considerations

The Human Research Ethics Committee of Universiti Tun Hussein Onn Malaysia waived the need for ethical approval. The participant was voluntary and gave verbal informed consent.

## 5. Performance of antenna and EBG on body

### 5.1 Reflection coefficient

**Fig 13:**
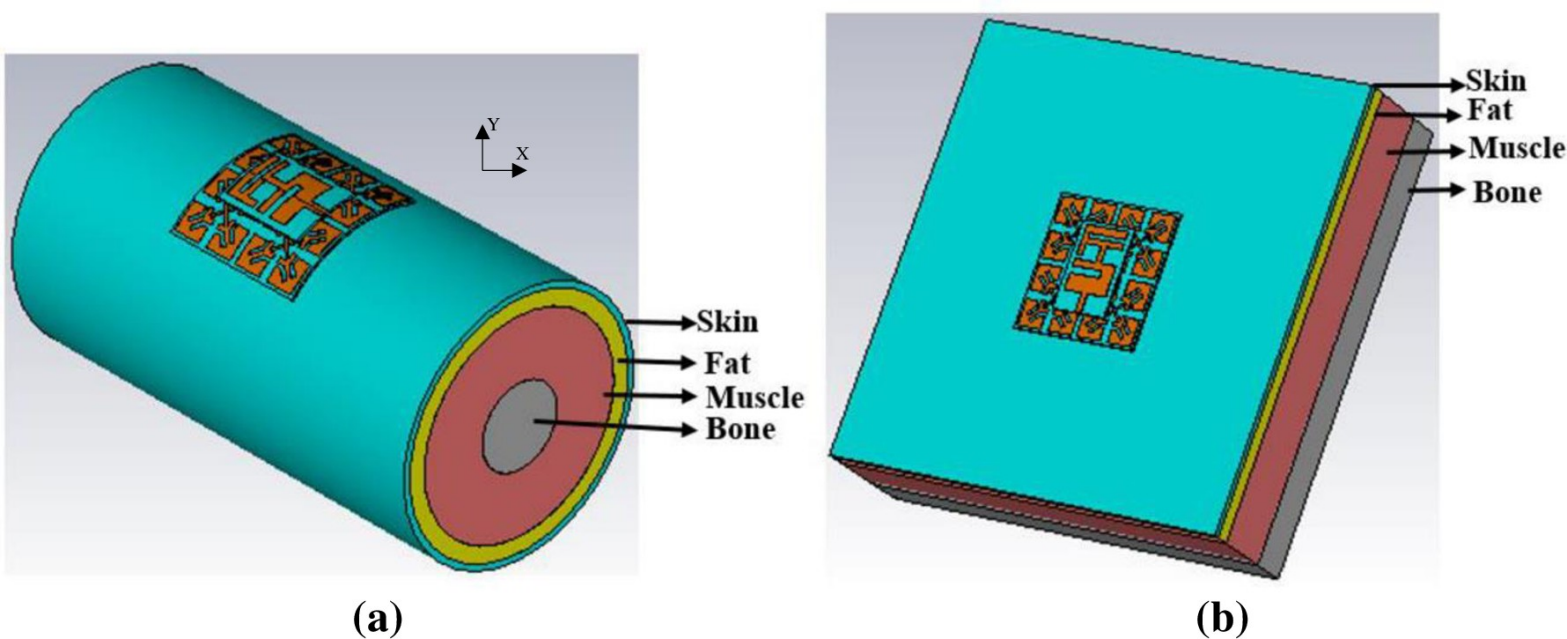
Developed human models (a) Arm, and (b) Chest.

The reference antenna and the modified antenna with EBG have been simulated and measured on the body to validate their operational performance due to the body's high dielectric. Two parts of the body, the chest, and the arm were chosen for the numerical and the experimental investigation. The selection of these two parts is to evaluate the proposed design in terms of bending (around the arm) and normal case (mounted over the chest). This is to ensure the functionality of the proposed design in both cases when loaded on human tissues. The models are developed using CST for numerical studies. The cylinder with a diameter of 80 mm and 150 mm length was developed to imitate the arm shape Fig 13(A) [5, 11]. A square shape with a length and width of 150 mm and a thickness of 40 mm was designed to emulate the chest shape as depicted in Fig 13(B). Each model consists of four layers: fat, bone, skin, and muscle, and their corresponding typical conductivity, thickness, mass density, and permittivity values for each layer are summarized in Table 4 [5, 11].

**Table 4:** Properties of the model tissue [5, 11].

	Bone	Muscle	Fat	Skin
Thickness (mm)	13	20	5	2
Density (kg/m3)	1008	1006	900	1001
*σ* (S/m)	0.82	1.77	0.11	1.49
*ε_r_*	18.49	52.67	5.27	37.95

**Fig 14:**
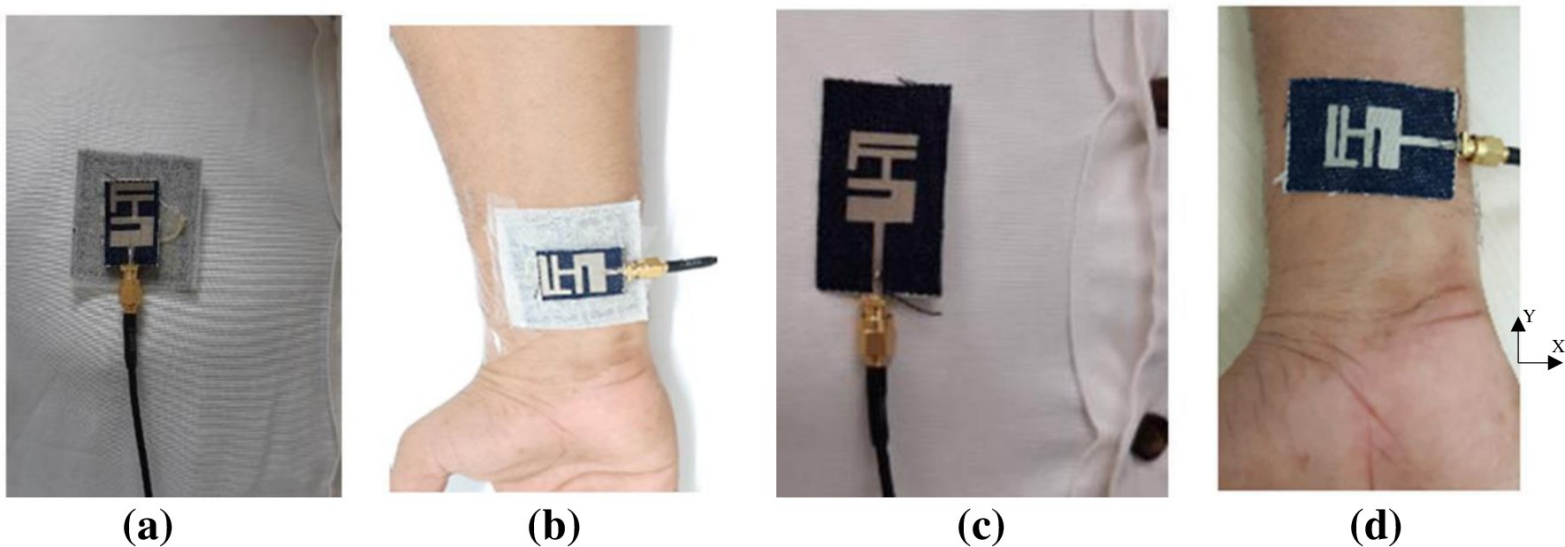
(a) Proposed design on chest, (b) Proposed design on arm, (c) Reference antenna on chest, and (d) Reference antenna on arm.

A volunteer male weighing 79 kg and 160 cm tall are used for experimental studies to verify the simulated performance. The design was bent on the arm and mounted on the chest, as shown in Fig 14. The arm has a diameter of 80 mm, which is equivalent to the developed arm. This study gives extra credibility to work by adopting a real human body instead of simulated models.

**Fig 15:**
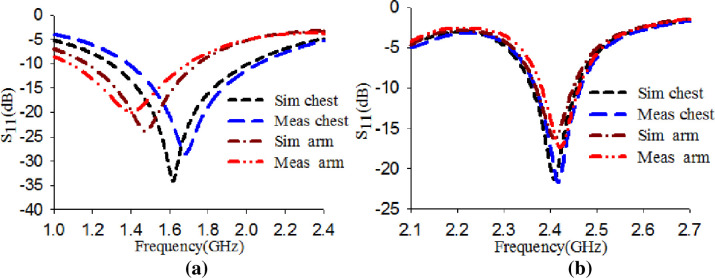
S_11_ performance of designs placing on the chest and the arm (a) Reference antenna and (b) Proposed design.

As shown in Fig 15(A), the reference antenna leads the S_11_ to shift from 2.4 GHz to a lower band for both arm and chest case. This is because the human body acts as an additional complex substrate layer that has high permittivity. Therefore, placing the reference antenna directly on the human body results in a severe impedance mismatch of the antenna. In the case of integrating the modified antenna with EBG, the required spectrum bandwidth is achieved as described in Fig 15(B). This is because the EBG shields the antenna from the body effects. The measurement results are almost identical to the simulation results. Hence, the modified antenna with EBG is a well-suitable candidate for wearable applications, including medical devices.

**Fig 16:**
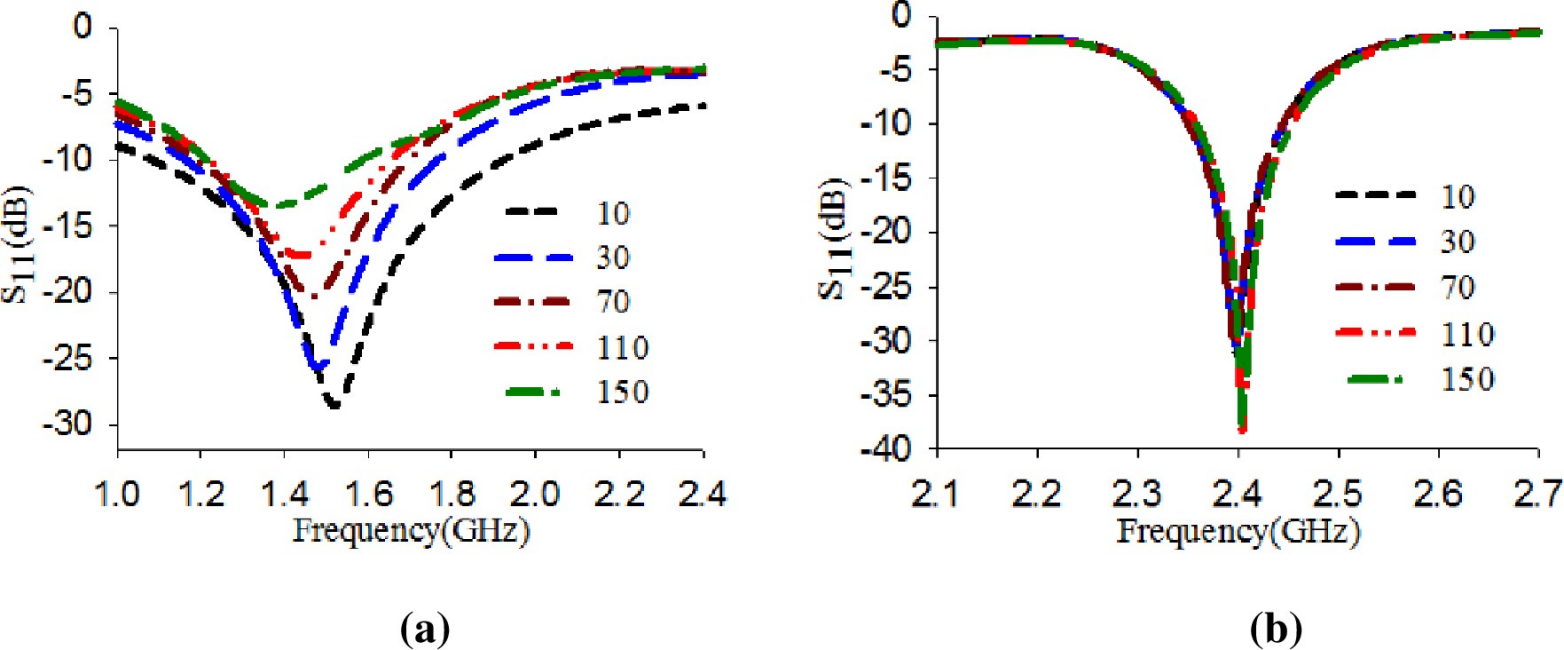
S_11_ performance with varying the dielectric constant of the skin layer (a) Reference antenna and (b) Proposed design.

Further investigation is conducted to analyze the worst-case scenario of varying the dielectric constant (ε_r_) of the human model tissue on the proposed design's performance in terms of its S_11_ stability. This investigation is carried out to ensure the proposed design's robustness and functionality when loaded on a high dielectric constant [39]. Therefore, we assume that the skin layer has values in the range of 10 to 150. The design was placed directly on the model. The S_11_ of the reference antenna and the modified antenna with EBG were obtained for different permittivity values (ε_r_), namely 10, 30, 70, 110, and 150, as shown in Fig 16. The results showed that when the antenna alone, the S_11_ was shifted to the lower band and did not operate at the desired band. Meanwhile, with EBG, the S_11_ is maintained at the desired band. This proves that the EBG act as isolation between the body and the antenna. It also demonstrates its robustness and functionality even if it loaded at a high dielectric constant.

### 5.2 Radiation patterns

**Fig 17:**
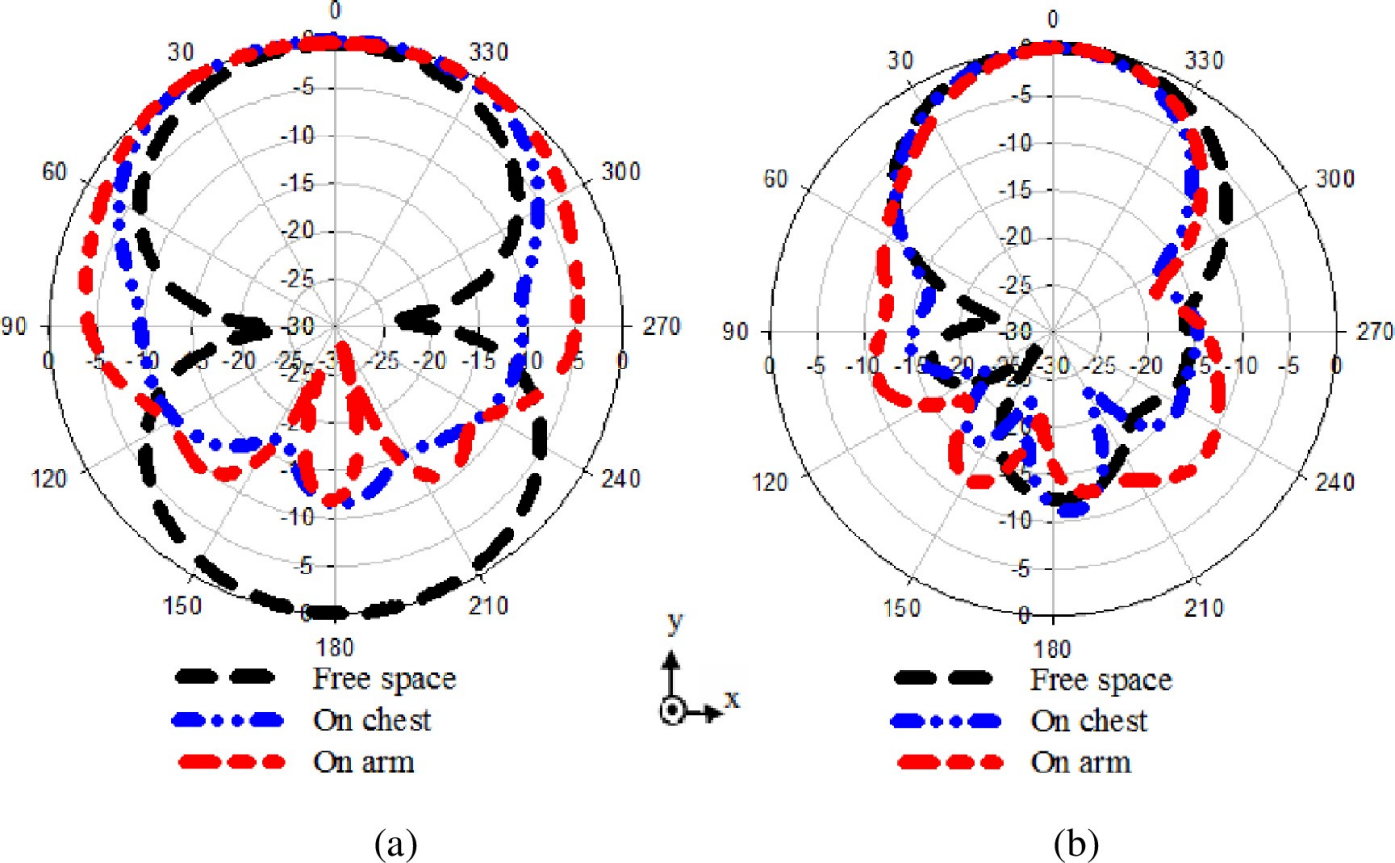
Radiation pattern performance placing on chest and arm (a) Reference antenna, and (b) Proposed design.

Fig 17 displayed the normalized radiation patterns’ performances when the antenna was loaded on the chest and arm. The result illustrates that the radiation properties of the reference antenna is greatly influenced and become more directive when placed on the human body because of its high permittivity compared to the fabric substrate of the reference antenna, as shown in Fig 17(A). This indicates that the body absorbed the power, which could cause a health risk while placing the modified antenna with EBG on the chest and arm reveal a reasonable radiation characteristic as shown in Fig 17(B). It can maintain its shape pattern as free space with the minor difference that does not affect the radiation performance.

### 5.3 SAR

SAR is a standard measure of power deposition in the human tissues per unit mass. Therefore, its assessment is vital to ensure that safety limits are met. The same model used in section 5.1 is used for numerical SAR analysis to verify the performance. According to the Federal Communication Commission (FCC), the 1 g averaged SAR value should not exceed 1.6 W/kg [39]. The following equation relates SAR with the applied input power used to calculate the SAR:

$$SAR=\frac{\sigma |E^{2}|}{\rho }$$ (1)

Where *ρ* is the mass density of the tissue in kg/m^3^, E is the electric field in V/m, and *σ* is the conductivity of the tissue in S/m.

The SAR evaluation is conducted using the IEEE C95.1 standard available in the CST MWS software. The input power for the calculation is taken as 100 mW [5, 11, 12]. Table 5 summarizes the SAR calculation results for both designs, taken for different scenarios, including cases where the antenna is mounted on the arm and the chest. The SAR calculation was also performed with antenna placements at 0 mm (touching the skin), 1 mm, and 3 mm away from the body part. The table shows that, for the antenna without EBG, the SAR value averaged over 1g decreased with increasing distance between the antenna and the phantom model. However, these SAR values still exceed safety limits governed by the FCC standard throughout all antenna placements.

**Table 5:** SAR (W/kg) values of proposed design.

Distance from the phantom (mm)	Antenna w/o EBG			Antenna w/EBG		
	On arm		On chest	On arm		On chest
	y-axis	x-axis		y-axis	x-axis	
0	13.6	11.2	7.41	0.132	0.050	0.058
1	6.05	5.98	5.7	0.054	0.036	0.037
3	5.22	5.7	4.36	0.047	0.025	0.028

**Fig 18:**
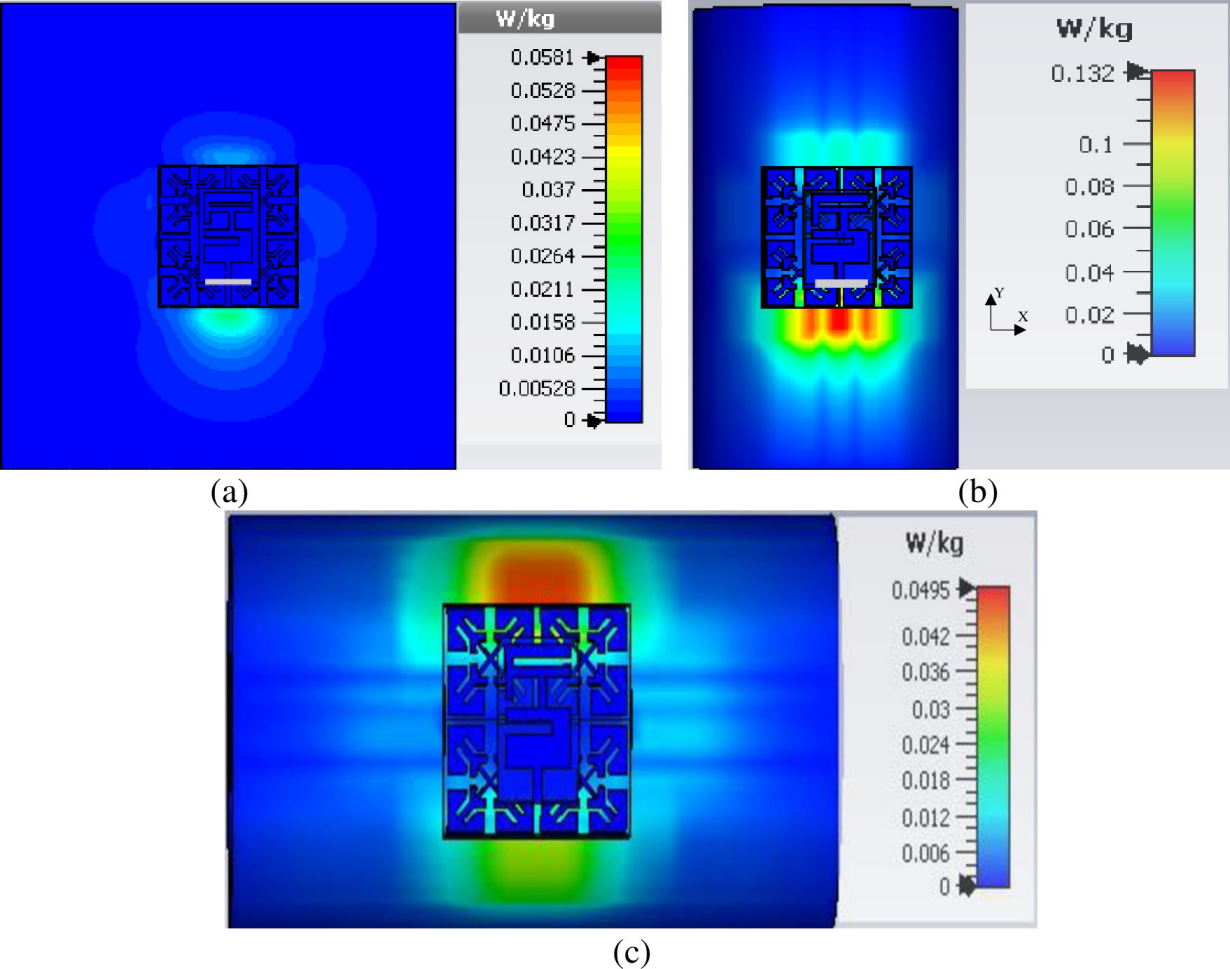
SAR values of proposed design when touch the skin over 1 g (a) On chest, (b) On arm y-axis, and (c) On arm x-axis.

Although the antenna was 3 mm distant from the human body, it still shows a maximum SAR of 5.22 W/kg averaged over 1g. This is due to its omnidirectional radiation characteristic [5, 11]. When integrated with EBG, a substantial drop in the maximum SAR value averaged over 1 g is realized, thus complying with the safety standards, even when the antenna integrated with EBG is directly placed on the skin (touching the skin) as shown in Fig 18. The reduction is more than 95% compared to the proposed reference antenna without the EBG, showing the superiority of the antenna when incorporated with the EBG.

The SAR also was evaluated by changing the skin layer electrical properties to analyses the worst-case scenario of SAR. The result of the antenna with and without EBG is tabulated in Table 6. It is observed that for all analyzed cases, namely for *ε_r_* = (10, 30, 70, 110, 150), when the antenna alone, the SAR values exceed the safety limit set by FCC. However, when integrating the EBG structure with the antenna, the SAR values are much lower than the FCC limits for all analyzed cases. These simulated results were compared with other similar published works as tabulated in Table 7. They all seem very close values to the literature. Table 7 depicts the comparison of the proposed wearable antenna design's performance with other backed two years of published works. As can be seen, the proposed design outperforms the other reported work based on overall size, FBR, gain, and comparable performance in terms of efficiency, SAR, and the unit cell number.

**Table 6:** SAR (W/kg) values of varying the dielectric constant of skin layer.

Permittivity Design	10	30	70	110	150
Antenna w/o EBG	11	10.1	8.17	8.95	6.89
Antenna w/EBG	0.037	0.030	0.022	0.017	0.016

**Table 7:** Comparison of previous work with proposed design based on flexible materials at 2.4 GHz.

Ref	Year	Efficiency (%)	Gain (dBi)	SAR W/kg	FBR (dB)	No. of unit cell	Overall size (mm^3^)	Substrate type
[29]	2018	71	7.3	0.230	17	3×3	81×81×4	Fabric
[21]	2019	49	8.3	-	-	-	70×85×6	Felt
[23]	2020	70.7	6.56	0.612	-	3×3	60×60×8.5	PDMS
[15]	2020	-	6.45	0.983	16	2×2	60×60×2.4	Fabric
[7]	2020	44.39	4.06	0.521	-	1×2	50×25.7×5	Felt
[30]	2020	-	1.94	0.111	12	3×3	85.5×85.5×5.28	Felt
[22]	2020	61	6.19	-	-	4×3	145×112×3.424	Jeans
[24]	2020	74.8	6.51	0.22	11.4	2×2	56×56×6	Ultralam850,Felt
This work	-	74.8	7.2	0.042	12.2	2×2	46×46×2.4	Fabric

## 6. Conclusion

A compact fully fabric antenna with a miniaturized EBG structure for wearable applications was successfully designed and experimentally tested. In contrast to earlier documented EBG backed antenna designs, the proposed EBG behaves as shielding from the antenna to the human body, reduces the size, and acts as a radiator. It also shows band-gap features without requiring vias. The miniaturized EBG array in the proposed design has contributed to enhancing the radiation efficiency and providing isolation between the antenna and human tissues. The antenna’s behavior under flexing and loading on a human body was experimentally investigated. The measured performance of the proposed antenna-EBG structure compares favorably to the most recent reported results. The experimental results show that the antenna-EBG integration achieves a gain of 7.2 dBi, an efficiency of 74.8%, and an FBR of 12.2 dB. Furthermore, the specific absorption rate (SAR) shows a reduction of more than 95% compared to the antenna alone and complies with the FCC standard. Hence, the presented incorporated antenna with EBGs is a well-suitable candidate for wearable applications, including medical devices.
